# Suppression of atom motion and metal deposition in mixed ionic electronic conductors

**DOI:** 10.1038/s41467-018-05248-8

**Published:** 2018-07-25

**Authors:** Pengfei Qiu, Matthias T. Agne, Yongying Liu, Yaqin Zhu, Hongyi Chen, Tao Mao, Jiong Yang, Wenqing Zhang, Sossina M. Haile, Wolfgang G. Zeier, Jürgen Janek, Ctirad Uher, Xun Shi, Lidong Chen, G. Jeffrey Snyder

**Affiliations:** 1 State Key Laboratory of High Performance Ceramics and Superfine Microstructure, Shanghai Institute of Ceramics, Chinese Academy of Sciences, Shanghai, 200050 China; 2 Department of Materials Science and Engineering, Northwestern University, Evanston, Illinois 60208 USA; 3 University of Chinese Academy of Sciences, Beijing, 100049 China; 4 Materials Genome Institute, Shanghai University, Shanghai, 200444 China; 5 Department of Physics, South University of Science and Technology of China, Shenzhen, 518055 China; 6 Institute of Physical Chemistry & Center for Materials Research, Justus-Liebig-University Giessen, Heinrich-Buff-Ring 17, 35392 Giessen, Germany; 7 Department of Physics, University of Michigan, Ann Arbor, 48109 USA

## Abstract

Many superionic mixed ionic–electronic conductors with a liquid-like sublattice have been identified as high efficiency thermoelectric materials, but their applications are limited due to the possibility of decomposition when subjected to high electronic currents and large temperature gradients. Here, through systematically investigating electromigration in copper sulfide/selenide thermoelectric materials, we reveal the mechanism for atom migration and deposition based on a critical chemical potential difference. Then, a strategy for stable use is proposed: constructing a series of electronically conducting, but ion-blocking barriers to reset the chemical potential of such conductors to keep it below the threshold for decomposition, even if it is used with high electric currents and/or large temperature differences. This strategy not only opens the possibility of using such conductors in thermoelectric applications, but may also provide approaches to engineer perovskite photovoltaic materials and the experimental methods may be applicable to understanding dendrite growth in lithium ion batteries.

## Abstract (web summary)

Mixed ionic–electronic conductors are limited by material decomposition. Here the authors reveal the mechanism for atom migration and deposition in Cu_2–*δ*_(S,Se) materials based on a critical chemical potential difference and propose electronically conducting, ion-blocking interfaces to enhance stability.

## Introduction

Superionic conductors are solids in which at least one type of atom forms a rigid sublattice framework and another type of atom forms a “melted” liquid-like sublattice composed of highly mobile charged atoms, i.e., ions^1^. The description of the ensemble of highly mobile ions in terms of a “liquid” sublattice derives from the fact that the entropy change during transition into the superionic phase is comparable to the entropy change during a solid/liquid phase transition^1^. This unique liquid-like ion migration is the basis for many phenomena and applications in the fields of solid-state ionics, e.g., of solid electrolytes, batteries, fuel cells, and various types of sensors^2^. Ion migration also occurs in materials with much lower ion mobility, and is often the origin of electric field-driven degradation in hybrid organic–inorganic perovskite solar cell materials (e.g., CH_3_NH_3_PbI_3_) and Cu-based photovoltaic materials^3,4^. Ion migration and the redox-based formation of conducting paths in dielectrics is the basis for future information storage technologies (atomic switching and memristive devices)^5^.

Recently, the application of superionic mixed ionic–electronic conductors (MIECs) with a liquid-like sublattice has been extended to the field of thermoelectrics (TEs). A concept named as “phonon-liquid electron-crystal” (PLEC) has been proposed to design and develop high-performance TE materials that conduct phonons like a liquid and electrons like a crystal^6^. A large family of novel Cu-, Ag- and Zn-based superionic MIECs have been identified satisfying this concept—with typical examples being Cu_2-*δ*_*X* (*X* = S, Se, Te), Ag_2_Se, CuAgSe, Zn_4_Sb_3_, Cu_5_FeS_4_, Cu_7_PSe_6_, and Cu_12_Sb_4_S_13_, etc^6–12^. In a MIEC, both the ions and electrons (or holes) are mobile such that atom (rather than ion) migration is possible, which may lead to composition changes within the MIECs^13^. Many new and unusual electrical and thermal transport properties, such as the reduced specific heat, ultralow and temperature independent lattice thermal conductivity, and extremely high TE figure of merit *zT* > 2.0 have been observed and reported in these MIECs^14^. The high TE performance and the fact that Cu is relatively non-toxic and earth-abundant has attracted much attention to these MIECs for both fundamental studies and industrial applications.

**Fig. 1:**
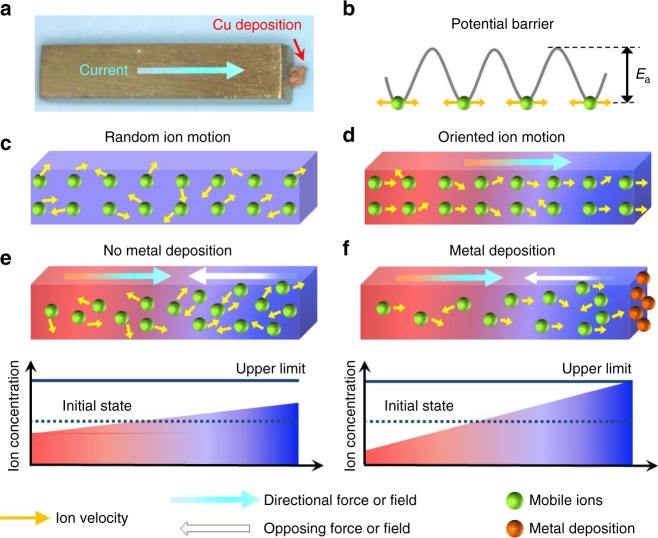
Atom migration and deposition in ion conductors. **a** Metallic Cu deposition on the surface of a Cu_2_S sample induced by a current. **b**–**e** Schematic of (**b**) the energy landscape for ions; **c** random ion motion without net flux; and **d** net ion flux under directional force or field. Due to a directional force or field and depending on the electrode constraints, MIECs may either reach a (**e**) steady state without net ion transport (and without metal deposition) or (**f**) continuous metal deposition (or other decomposition), if the local Cu concentration reaches a critical level determined by the stability range of the MIECs

Despite the high performance, the stability and reliability of MIECs are key concerns for long-term service in real applications for batteries, photovoltaics, and TEs. In Li-ion batteries, for instance, Li dendrite growth can result in catastrophic failure leading to combustion or explosion^15^. In CdTe–Cu_*x*_Te solar cells, Cu atoms can migrate into the CdTe absorber and CdS window layers to form carrier traps that reduce device performance^4^. In TEs, the added potential risk using MIECs contributed to the discontinuation of Cu_2–*δ*_Se development for aeronautics and space applications^16,17^. The mobile Cu species in Cu-based MIECs are prone to deposit on the surface at the cathode to form Cu metal when an external electric field or temperature gradient is applied (Fig. 1a). If Cu is not supplied at the anode, the Cu metal deposition at the cathode will change the initial material’s composition and degrade TE performance as the metal deficiency in MIECs is optimized for a high *zT*. In TE devices with MIECs, Cu metal deposition on the sample surface may also damage the contact between the TE material and the electrode by forming cracks to increase electrical/thermal resistance and degrade the power output and conversion efficiency^18^. In addition, the plating of Cu at the cathode may lead to the evaporation of chalcogenides at the anode, resulting in the breakage of the thermolegs^19^. This is partly why NASA stopped the program of Cu_1.97_Ag_0.03_Se_1+*y*_-based radioisotope thermal generators in 1981 after more than 10 years of research activity^18^. Therefore, metal deposition caused by ion flux must be restricted to improve the stability and reliability of TE devices consisting of MIECs before the use in any industrial application.

In this study, through systematically investigating the behavior of various MIECs with a liquid-like ionic sublattice in an electric field with or without a temperature gradient, we reveal the relations of ion migration, metal deposition, and materials degradation in MIECs for TE application. A general model is proposed to reveal the thermodynamic threshold for decomposition of MIECs. With this understanding, we develop a strategy to improve the MIECs’ stability and reliability by adding electronically conducting, but ion-blocking interfaces in the material.

## Results

### Mechanism of ion transport in mixed conductors

The physical and chemical processes of atom migration and metal deposition in MIECs are presented in Fig. 1b–f. The mobile ions (Cu^+^ in Cu_2_S and Cu_2_Se) possess high diffusivities, as they have relatively low activation energies for migration, *E*_a_ (e.g., 0.19 eV in Cu_2_S and 0.14 eV in Cu_2_Se)^20,21^. Simply, neighboring sites are energetically close and jumps are frequent (Fig. 1b). If there is no directional force or field applied to the uniform material, the random motion of ions is equal in all directions and there will be no net mass transport in a specific direction (Fig. 1c). In response to an external driving force (an electric field or temperature gradient) the charge carriers (ions and electronic species) flow (Fig. 1d). If the electrodes do not allow for ion transfer, there can be no net ionic current in the stationary state, and a concentration gradient of ions will form within the material. For reasons of electroneutrality, this concentration gradient will lead to an equal concentration gradient of electrons, and in essence, a chemical composition gradient is formed. If the concentration variation remains within the stability limits of the material, i.e., within the homogeneous phase field defined by the phase diagram of the MIEC, the concentration gradient of the mobile component acts as diffusion force opposing the external applied force on the mobile component, and a steady-state condition is achieved, i.e., there is no driving force for further migration of the neutral component (Fig. 1e). In the following, we describe transport of the neutral metal component as transport of atoms for the sake of simplicity. In the steady-state condition with ion-blocking electrodes, the material can still transport a net flux of electric charge (via electrons or holes in the presence of an electric field) and heat (via electrons or holes and phonons in the presence of a temperature gradient), but the net flux of atoms is zero. This steady-state condition of vanishing atom transport, also called the Soret steady state^22^, is similar to the condition in a conventional TE material (e.g., Bi_2_Te_3_, PbTe, and SiGe)^23–25^.

However, the change in the chemical potential of the migrating atom may lead to decomposition of the MIEC and prevent the formation of a steady-state condition. At the electrode interface, a critical chemical potential may be reached where a decomposition (product) phase is favored. For instance, in Cu_2-*δ*_(Se,S), when the chemical potential of Cu at the cathode is equal to or higher than the chemical potential of Cu metal, the reduction of Cu^+^ to Cu metal at the cathode or the oxidation of selenium/sulfur anions to Se/S (solid and/or gaseous) at the anode can occur^16–19^. The chemical potential beyond which decomposition occurs also corresponds to a “solubility limit” of Cu in the MIEC. The maximum solubility of Cu precedes Cu metal deposition and the minimum solubility of Cu precedes Se/S oxidation. When the Cu concentration increases beyond this “solubility limit”, Cu metal will deposit at the cathode if the kinetics of metal crystallization allows (Fig. 1f). Once this happens, the material and the interface in the device can be permanently altered. We note that the deposition of Cu metal is hindered by a nucleation barrier. Then, for Cu deposition to occur, the chemical potential of Cu needs to be slightly higher than its standard potential, *µ*°(Cu), but all the general conclusions above remain the same.

The thermodynamic threshold for the maximum or minimum solubility in Cu-based MIECs can be reached if there is a sufficient change in the chemical potential of Cu atoms due to applied forces (electric field and temperature difference). In steady state, the net change in chemical potential of Cu atoms can be determined from the sum of the change in electrochemical potentials of the constituent ions and electronic carriers. Because copper atoms are in equilibrium with copper ions and electrons, the electrochemical potentials $\left( {\tilde \mu } \right)$ are related by $\mu _{\mathrm{Cu}} = \tilde \mu _{\mathrm{Cu}^ + } + \tilde \mu _{\mathrm{e}}$. Thus the change in chemical potential across the material (defined by the electrodes each end is in contact with),

1 $$\Delta \mu _{{\mathrm{Cu}}} = \mu _{{\mathrm{Cu}}}^{{\mathrm{anode}}} - \mu _{{\mathrm{Cu}}}^{{\mathrm{cathode}}} = \Delta \tilde \mu _{{\mathrm{Cu}}^ + } + \Delta \tilde \mu _{\mathrm{e}}$$

Consequently, our goal is to relate experimental parameters to *μ*_Cu_ in order to explain the critical condition of Cu metal deposition in Cu-based MIECs.

From the flux equation of linear nonequilibrium thermodynamics (see Supplementary Note 1)^26,27^, the electronic current density, *J*, is driven by the gradient of the electrochemical potential of the electronic species and the temperature gradient through the relation

2 $$J = - \sigma \left[ {\frac{1}{{z_{\mathrm{e}}F}}\nabla {\tilde{\mu}}_{\mathrm{e}} + S_{\mathrm{e}}\nabla T} \right]$$

where *z*_e_ defines the charge (−1 for electrons or +1 for holes), *F* is Faraday’s constant, and *σ* is the specific electrical conductivity. *S*_e_ which has the sign and units of the electronic Seebeck coefficient, captures the effect of thermodiffusion due to the temperature gradient ∇*T*. Using the analogous flux equation for ions, considering the case when ion-blocking electrodes are used (i.e., there is no ion flux, $J_{{\mathrm{Cu}}^ + } = 0$), then the electrochemical potential driving force exactly cancels the thermal driving force for ion migration. Explicitly,

3 $$\nabla \tilde \mu _{{\mathrm{Cu}}^ + } = - z_{{\mathrm{Cu}}^ + }F \cdot S_{{\mathrm{Cu}}^ + }\nabla T$$

and $S_{{\mathrm{Cu}}^ + }$ similarly relates to the thermodiffusion of Cu ions. When the gradients in Eqs. (2) and (3) only apply in the *x* direction they can be integrated, which for linearly varying systems (such as MIECs^28^) is equivalent to being multiplied through by Δ*x* = *L* (*L* is the effective length between electrodes), and used in the relation defined by Eq. (1). Upon rearranging, we arrive at

4 $$V = \frac{{JL}}{\sigma } = - \frac{1}{{z_{\mathrm{e}}F}}{\mathrm{\Delta }}\mu _{{\mathrm{Cu}}} - S^ \ast {\mathrm{\Delta }}T$$

where S^*^ accounts for the net effect of thermodiffusion and *V* is the experimental parameter (in units of voltage, calculated from *J*) of this study.

Because there are bounds to the chemical potential range over which the material can exist, there must be some critical chemical potential difference $\left( {{\mathrm{\Delta }}\mu _{{\mathrm{Cu}}}^{{\mathrm{crit}}}} \right)$ at which Cu metal deposition occurs. From Eq. (4), it is straightforward to find the voltage corresponding to this critical chemical potential difference:

i. in the isothermal case,
  5 $$V_{\mathrm{c}} = - \frac{1}{{z_{\mathrm{e}}F}}{\mathrm{\Delta }}\mu _{{\mathrm{Cu}}}^{{\mathrm{crit}}}$$
  where *V*_c_ is a critical voltage that results from a critical applied current density *J*_c_, and,
ii. in a temperature difference,

6 $$V_{\mathrm{c}} = - \frac{1}{{z_{\mathrm{e}}F}}{\mathrm{\Delta }}\mu _{{\mathrm{Cu}}}^{{\mathrm{crit}}} - S^ {\ast} {\mathrm{\Delta }}T$$

where it is important to note that the sign of Δ*T*(=*T*_anode_–*T*_cathode_) is important, as will be discussed.

From this analysis it is expected that a voltage difference, not current density, is the critical parameter for Cu deposition. We also note that in open circuit conditions (no electronic or ionic current) the thermodynamics predicts a critical Δ*T* resulting in Cu deposition at the cathode (when *V*_c_ = 0 in Eq. 6).

Further consideration of the isothermal condition (Eq. 5) reveals that *V*_c_ only depends on the composition of the compound relative to the “solubility limit”. This allows for the use of a microscopic defect model to relate off-stoichiometry, *δ*, (in Cu_2-*δ*_*X*, *X* = S, Se) to the critical chemical potential in Cu-based MIECs. A parameter named as the critical off-stoichiometry (*δ*_c_) is introduced here, corresponding to the “solubility limit” of Cu concentration at the cathode of the MIEC. Based on the theory proposed by Yokota and Korte et al.^28,29^,

7 $$V_{\mathrm{c}} = - \frac{{RT}}{F}\left( {{\mathrm{Arsinh}}\left( {\frac{{\delta _{\mathrm{c}}}}{{2\sqrt {K_{\mathrm{e}}} }}} \right) - {\mathrm{Arsinh}}\left( {\frac{{2\delta - \delta _{\mathrm{c}}}}{{2\sqrt {K_{\mathrm{e}}} }}} \right)} \right)$$

where *K*_e_ is the equilibrium constant for electrons and holes that is independent of stoichiometry, *R* is the gas constant, and *T* is the temperature. The thermodynamic theory developed herein predicts that a given off-stoichiometry and temperature difference will result in limitations on the electrical potential difference that is stable across the material. Using this knowledge, the latter part of this paper will address possible ways to engineer stability in these materials for TE applications.

### Isothermal ion transport in mixed conductors

**Fig. 2:**
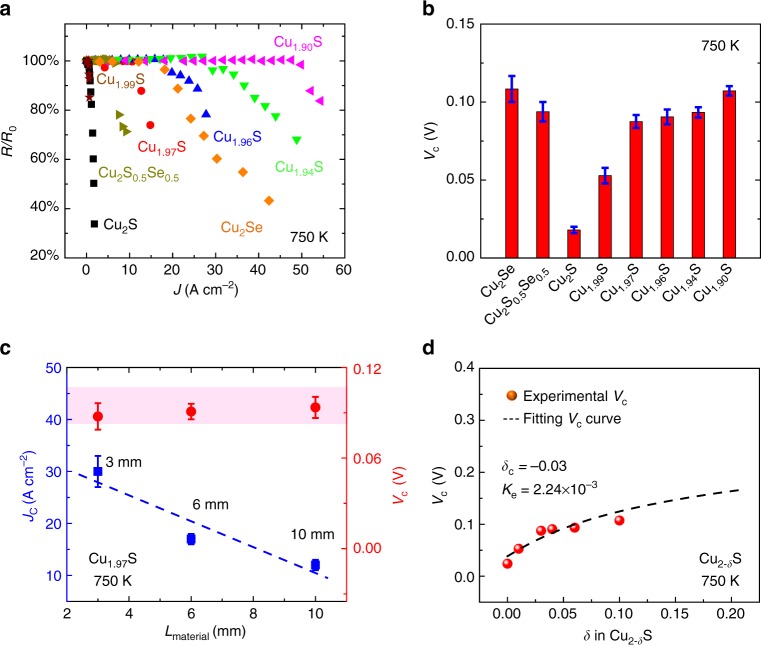
Critical electric potential difference (*V*_c_) in the isothermal case. **a** Current density dependence of relative electrical resistance variation (*R*/*R*_0_) for several Cu-based mixed ionic/electronic conductors with *L* = 10 mm. **b** Experimentally determined *V*_c_ (*L* = 10 mm). **c** Material length *L* dependences of *V*_c_ and the critical current density *J*_c_ for Cu_1.97_S. The dashed line is a guide to the eyes. **d** *V*_c_ as a function of Cu off-stoichiometry *δ* in the Cu_2-*δ*_S (*δ* *=* 0, 0.01, 0.03, 0.04, 0.06, and 0.1) samples with *L* = 10 mm. The dashed line represents the *V*_c_ curve based on Eq. 7. All measurements were carried out at 750 K

Taking the family of Cu-based TE MIECs as an example, the determination of *V*_c_ is introduced for the isothermal case. When an externally applied electric field does not raise *V* between the two ends of the sample to the critical *V*_c_ value, the Cu atoms simply redistribute inside the sample to form a steady-state concentration gradient of Cu without metal deposition at the cathode (Fig. 1e). After removing the electric field, the Cu concentration gradient gradually returns to the initial homogeneous state (Supplementary Fig. 1). Thus, for *V* *<* *V*_c_, the Cu redistribution under the external electric field is temporary, with no lasting effect on the MIEC. However, when the external electric field reaches *V*_c_ (Fig. 1f), Cu metal can  deposit. Although metallic Cu is not thermodynamically stable after the electric field is removed, many of the metallic deposits cannot immediately diffuse back into the sample (kinetically limited). In this case, the average Cu concentration inside the MIEC is reduced (Supplementary Fig. 1), which causes measureable changes of the resistivity (Fig. 2a).

The *V*_c_ values for several Cu-based TE MIECs, including Cu_2–*δ*_S (*δ* *=* 0, 0.01, 0.03, 0.04, 0.06, and 0.1), Cu_2_Se, and Cu_2_S_0.5_Se_0.5_, are experimentally determined at a constant temperature of 750 K (Supplementary Table 1) by using the apparatus and method shown in Supplementary Figs. 2, 3. The details can be found in the Supplementary Methods. The maximum *V*_c_ value for all samples is only 0.11 V (Fig. 2b). Such small values are consistent with the ease of observing Cu-metal deposition in these TE MIECs^16,17^. Notice that *V*_c_ is constant for a series of Cu_1.97_S samples with various material lengths, *L*, whereas *J*_c_ decreases with increasing *L* (Fig. 2c). This agrees well with Eqs. (4–7) in which *V*_c_ is not dependent on length but *J*_c_ is. Thus, although it may seem natural to be concerned about metal deposition due to high current density in a TE generator device, it is the voltage and not the current density that defines the critical quantity, which is in agreement with the insight of Eq. 4.

Furthermore, we found experimentally (Fig. 2d) that *V*_c_ gradually increases with increasing Cu off-stoichiometry (*δ* in Cu_2-*δ*_S). Intuitively, this coincides with a reduction in the chemical potential of Cu as the off-stoichiometric material is more willing to accept Cu atoms. This trend can be well explained by Eq. (7). According to the Cu–S binary equilibrium phase diagram^30^, Cu_2-*δ*_S has a wide composition range (0 < *δ* *<* 0.27) at 750 K. Thus, for these Cu_2-*δ*_S (*δ* = 0, 0.01, 0.03, 0.04, 0.06, and 0.1) samples, constant values for *δ*_c_ (=−0.03) and *K*_e_ (=2.24 × 10^−3^) fit the experimental data well using Eq. (7) (Fig. 2d). In theory, *δ*_c_ should correspond to the Cu-rich phase boundary composition found on the phase diagram, but here it is used as a phenomenological constant. Since *K*_e_ = *x*_n_*x*_p_ = exp(−Δ*G*_e_/*RT*), where *x*_n_ and *x*_p_ are the molar fractions of intrinsic electrons and holes, respectively^29^, the electron–hole pair free energy of formation, Δ*G*_e_, is estimated to be 0.4 eV; consistent with the band gap of cubic Cu_2_S reported by Lukashev et al.^31^ In addition, it was found experimentally that the *V*_c_ values for both Cu_2_S and Cu_2_Se increase with increasing temperature (Supplementary Fig. 4), which corresponds to an increase in ${\mathrm{\Delta }}\mu _{{\mathrm{Cu}}}^{{\mathrm{crit}}}$ associated with the increase in phase width of these MIECs with temperature^30,32^.

### Ion transport of mixed conductors in temperature difference

In the non-isothermal case, the relative directions of the electric current and the heat flux are expected to have dramatic effects on *V*_c_ (Eq. 6). If the current direction is the same as the heat flux direction (i.e., *T*_anode_ *>* *T*_cathode_), the electrical potential and temperature difference work together to drive atom migration to the cathode. Conversely, when the direction of the electric current is opposite to the heat flux (i.e., *T*_cathode_ > *T*_anode_), the current and temperature difference have opposed driving forces for atom migration. Although the necessary condition for metallic Cu to plate out is that the chemical potentials of Cu in the MIEC are that of Cu metal at the cathode, this is a necessary but not sufficient condition because some degree of overpotential may be required to initiate and drive the electrodeposition reaction. The rate of deposition is not addressed in the thermodynamics analysis here.

Nevertheless, irrespective of overpotential effects, ${\mathrm{\Delta }}\mu _{{\mathrm{Cu}}}^{{\mathrm{crit}}}$ is expected to be constant for a given temperature difference, regardless of the relative flux directions. This is because the range of chemical potentials corresponds to the range in compositional phase space, which is negligibly impacted by the electric field. For engineering applications, this allows us to contrast the critical current densities that can be applied relative to the temperature difference. For a constant magnitude of |*∆T*| and ${\mathrm{\Delta }}\mu _{{\mathrm{Cu}}}^{{\mathrm{crit}}}$ in Eq. (6), we can write

8 $$V_{{\mathrm{c}},{\mathrm{same}}} = J_{{\mathrm{c}},{\mathrm{same}}}L{\mathrm{/}}\sigma _{{\mathrm{avg}}} = - \frac{1}{F}{\mathrm{\Delta }}\mu _{{\mathrm{Cu}}}^{{\mathrm{crit}}}-S^ \ast |\Delta T|$$

when the current is applied in the same direction as the temperature gradient (*J*_c*,*same_), or

9 $$V_{{\mathrm{c}},{\mathrm{opposite}}} = J_{{\mathrm{c}},{\mathrm{opposite}}}L{\mathrm{/}}\sigma _{{\mathrm{avg}}} = - \frac{1}{F}{\mathrm{\Delta }}\mu _{{\mathrm{Cu}}}^{{\mathrm{crit}}} + S^ {\ast} |\Delta T|$$

when the current is applied in the opposite direction as the temperature gradient (*J*_c,opposite_). Here, *L* and *σ*_avg_ are the effective length and average electrical conductivity across the superionic phase on the sample, respectively. Put another way, *V*_c,same_ and *V*_c,opposite_ are different from the isothermal case due to the additional potential that results from the thermodiffusion of charged species (generalized Seebeck effect).

**Fig. 3:**
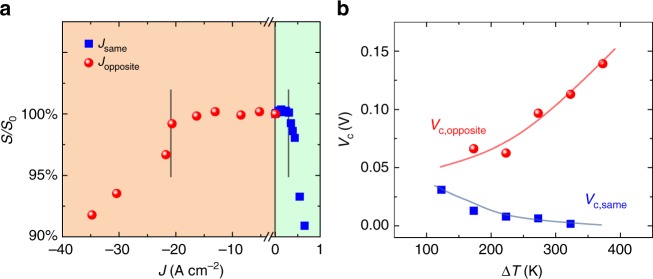
Critical electric potential difference (*V*_c_) under a temperature gradient. **a** The relative Seebeck coefficient variation (*S*/*S*_0_) as a function of the current density for Cu_1.97_S at *T*_cathode_ *=* 300 K and *T*_anode_ = 673 K. The positive *J* means that the current direction is the same as the heat flux direction. The negative *J* means that the current direction is opposite to the heat flux direction. **b** Experimentally determined *V*_c,same_ and *V*_c,opposite_ as a function of |*∆T*|, showing qualitative agreement with Eqs. 8 and 9.  The temperatures at the anode and cathode for each flux direction can be found in text. The length of all measured samples is 6 mm. The dashed lines are guides to the eye

Taking Cu_1.97_S as an example, the critical current density in the non-isothermal case is measured by using the apparatus and method shown in Supplementary Fig. 5. The details can be found in the Supplementary Methods. At a constant |Δ*T|*(= 673–300 K), a significant difference in critical current density is required depending on the relative flux directions (Fig. 3a). If the current direction is the same as the heat flux direction, *J*_c,same_ ≈ 0.3 A cm^−2^ is large enough to obtain metallic Cu deposition near the cold side. However, if the current direction is reversed, *J*_c,opposite_ ≈ 20 A cm^−2^ is required to obtain metallic Cu deposition at the hot side. This observation is in agreement with the thermodynamic expectations, although overpotential effects may contribute. To further demonstrate the trend predicted by Eqs. 8 and 9 , *V*_c,same_ and *V*_c,opposite_ were evaluated as a function of increasing |*∆T*| by using the *σ*_avg_ data shown in Supplementary Table 2 (Fig. 3b). As expected, *V*_c,opposite_ is found to increase with |*∆T*| and *V*_c,same_ is found to decrease with |*∆T*| (Fig. 3b).

### Strategy to improve stability in mixed conductors

In real TE generators the direction of the current flowing through the p-type legs is always the same as the direction of the heat flux (Supplementary Fig. 6). Consequently, the thermodynamic understanding validated through the temperature difference experiments is very helpful to design stable TE devices based on these high performance MIECs. Eq. 4 and Fig. 2c clearly show that *V*_c_ is length independent; thus, *V*_c,same_ in Eq. 8 is length independent as well. Changing the geometry cannot change the critical chemical potential—it is fixed for a given temperature, temperature gradient, and off-stoichiometry. However, the total voltage across a TE leg can be increased by using a series connection of several segments of MIEC material, in which electrically conducting, but ion-blocking interfaces are used between the individual segments. The reason for this is simple: the total voltage across the series-segmented leg, *V*_leg_, is the sum of voltages across each segment, *V*_seg_. When *n* segments are approximately the same length, and each segment has the same critical voltage, *V*_seg,c_, the critical voltage for the entire leg is *V*_leg,c_ = *n V*_seg,c_.

**Fig. 4:**
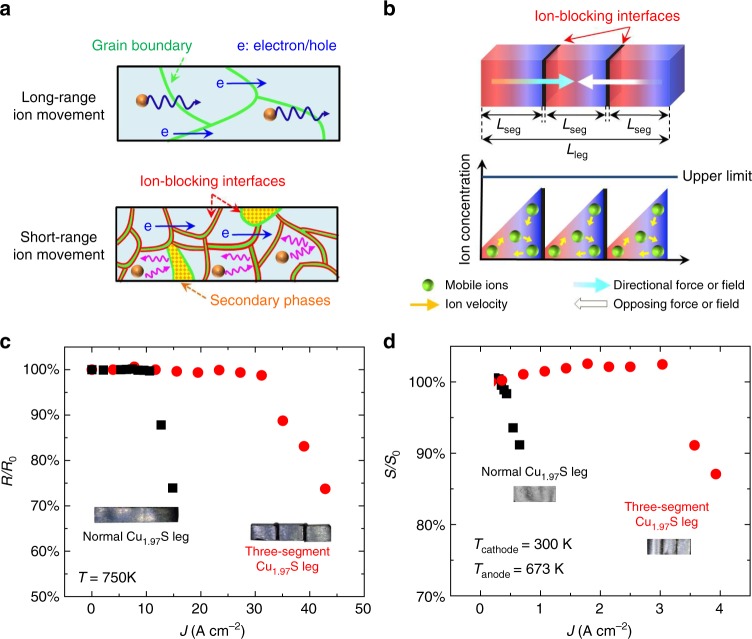
Ion-blocking strategy to improve stability in thermoelectric ion conductors. **a** Schematic for limiting the ion movement by including thin electron-conducting and ion-blocking interfaces; either grain boundaries (red areas) or a secondary phase (yellow areas). **b** Schematic of ion-blocking electrically conducting interfaces that allow the concentration profile to be reset at each interface so that the ion concentration does not ever reach the upper limit. **c** Relative resistance variation (*R*/*R*_0_) as a function of current density for different Cu_1.97_S samples at a constant temperature of 750 K without a temperature difference. **d** Relative Seebeck coefficient variation (*S*/*S*_0_) as a function of current density for different Cu_1.97_S samples under the condition of temperature difference (*T*_anode_ = 673 K and *T*_cathode_ = 300 K). The insets in (**c**) and (**d**) show the optical images of the measured Cu_1.97_S samples. The critical current density was measured across the segment in the middle

The schematic of this strategy is shown in Fig. 4a. These interfaces limit the ion movement but allow the free movement of the electrons or holes. Schematically, the ion concentration distribution rises linearly (as in Fig. 1e) in each segment, but because the chemical potential is reset by the ion-blocking interfaces a saw-tooth like pattern can be obtained. Engineering the number of segments *n* can allow for high voltages (and corresponding current densities) without reaching the critical chemical potential that results in the degradation of the material.

Initially, we test this strategy at a constant temperature of 750 K. The unsegmented Cu_1.97_S leg exhibits a critical applied current density of ≈11 A cm^−2^. However, for a three-segment Cu_1.97_S leg, the critical applied current density is ≈30 A cm^−2^ (Fig. 4c). This is in excellent agreement with the thermodynamic theory. The critical chemical potential for Cu at 750 K is then equivalent to *V*_seg,c_ = 0.09 V, as determined from the unsegmented leg. Because *V*_c_ is geometry independent, each segment has this same critical voltage. When the three segments are connected in series the critical voltage across the leg rises to *V*_leg,c_ = 0.27 V, exactly as expected.

Series segmentation is also effective in the non-isothermal case. As before, the critical voltage for the leg is the sum of critical voltages for each segment. However, the temperature dependencies of ${\mathrm{\Delta }}\mu _{{\mathrm{Cu}}}^{{\mathrm{crit}}}$ and S* may lead to different critical voltages for each segment. Nevertheless, the unsegmented leg can only substantiate a small current density of *J*_c,same_ = 0.3 Acm^−2^ (Fig. 4d). A three-segment leg, was found to sustain a significantly larger current density, *J*_c,same_ ≈ 3.0 A cm^−2^, about one order of magnitude higher than the unsegmented Cu_1.97_S leg (Fig. 4d). Correspondingly, the critical voltage across the leg is increased from *V*_c,same_ = 0.002 V to about 0.018 V. If more Cu-atom blocking layers are added, the critical total voltage across the leg can be expected to continue to increase. Therefore, the present data strongly suggests that MIECs can indeed sustain large current densities and achieve high stability whenever the local chemical potential is engineered to be lower than the corresponding critical chemical potential.

## Discussion

By understanding the thermodynamic principles behind ion and atom migration and metal deposition in TE MIECs, we derive a thermodynamic model to understand the critical electrical potential difference for decomposition of non-stoichiometric MIECs at constant temperature, and demonstrate that it is a critical voltage, not current, that is the limiting factor. In the more complicated case of concurrent electrical and thermal fluxes, the experimental observations are in excellent qualitative agreement with the thermodynamic predictions. Furthermore, we propose the use of ion-blocking but electrically conducting interfaces to enhance the critical electric potential difference in engineering applications. This is most efficiently done with a grain-boundary engineered microstructure. This study clearly shows that ion migration and metal deposition can be effectively suppressed in MIECs, which has been an overwhelming concern, but not solved before. Consequently, the technique demonstrated herein to increase the critical potential opens a new possibility of using these TE MIECs in real applications. We expect that the mechanism and strategies proposed in this study for TE materials should also be valid for other ionic conductors, and thus can be used in the research areas of, photovoltaics, solid electrolytes, and various sensors. Although the strategy of blocking ion transport is not applicable for batteries, the experimental and theoretical methods for characterizing and understanding atom deposition in MIECs should be applicable to Li batteries.

## Methods

### Materials synthesis

The detailed preparation process of the Cu-based TE MIECs used in the present study can be found elsewhere^14^.

### Critical voltage determination

The experimental parameter, *V*_c_, was ascertained by monitoring the electronic properties (e.g., electrical resistance *R* and Seebeck coefficient *S*) of the material after applying different electric currents or temperature gradients. Because the TE properties of the MIEC, especially the electrical resistance *R* and (electronic) Seebeck coefficient *S*, are very sensitive to the chemical composition, by monitoring the variation of the relative electrical resistance (*R*/*R*_0_, where *R*_0_ is the initial electrical resistance) or relative Seebeck coefficient (*S*/*S*_0_, where *S*_0_ is the initial Seebeck coefficient) under different current densities, the critical *V*_c_ can be determined. The critical current density, *J*_c_, corresponds to the point when *R*/*R*_0_ (or *S*/*S*_0_) begins to decrease is shown in Fig. 2a.

In the isothermal case, the chemical potential of Cu atoms is determined by the voltage on the sample generated by the electric current (see Eq. 4), which is determined experimentally as $V = \frac{{JL}}{\sigma }$, where *J* is the applied electric current density, and σ and *L* are the electrical conductivity and effective length of the MIEC, respectively. In the evaluation of *V*_c_, the temperature is set 750 K. The length of the measured sample is 10 mm. The typical 4-point electrical conductivity at the experimental temperature is used for resistance measurement. More measurement details can be found in Supplementary Methods.

In the non-isothermal case and *T*_anode_ > *T*_cathode_ (Eq. 6), the electrical potential and temperature difference work together to drive atom migration to the cathode. In this case, the Cu metal deposition will occur at the superionic-phase/normal-phase interface if *T*_cathode_ is lower than the superionic phase transition temperature of the MIEC; or, at the cathode if *T*_cathode_ is above the superionic phase transition temperature. When the relative fluxes are opposed (*T*_cathode_ is the hot side), Cu metal deposits at the cathode so long as *T*_cathode_ is above the superionic phase transition temperature. This was accounted for in the effective length, *L*, used to calculate *V*_c_. In the evaluation of *V*_c,same_, the cathode temperature *T*_cathode_ was fixed at 300 K, but |*∆T*| was calculated relative to the superionic phase transition temperature of Cu_1.97_S (≈ 350 K). The anode temperatures, *T*_anode_, were 473, 523, 573, 623, and 673 K. To determine *V*_c,opposite_, *T*_anode_ was fixed at 300 K and *T*_cathode_ was set to 473, 523, 573, 623, and 673 K. The length of the measured sample is 6 mm.

### Segmented leg construction

The *J*_c_ and *V*_leg,c_ values for a *n* = 3 segmented Cu_1.97_S leg (total length is *L*_leg_ = 10 mm) are experimentally obtained. The leg is made by bonding three *L*_seg_ ≈ 3.3 mm pieces together by using conductive carbon paste as the Cu-atom blocking layer. The data of this three-segment Cu_1.97_S leg are compared with those of the unsegmented Cu_1.97_S leg (*L*_leg_ = *L* = 10 mm). Figure 4d shows the relative Seebeck coefficient variation *S/S*_0_ values for a three-segment Cu_1.97_S leg with *L*_seg_ = 2 mm, *T*_anode_ = 673 K, and *T*_cathode_ = 300 K. The leg was fabricated in the same manner previously described. The data for the unsegmented Cu_1.97_S leg (*L*_leg_ = *L* = 6 mm) is included for comparison.

### Data availability

All data are available from the authors upon reasonable request.

The raw data contained in Figs. 2, 3, 4 and Supplementary fig. 4 are available upon request from X.S.

## Electronic supplementary material

Supplementary Information

## References

[1] Boyce JB, Huberman BA (1979). Superionic conductors: transitions, structures, dynamics. Phys. Rep..

[2] Knauth P, Tuller HL (2002). Solid-state ionics: roots, status, and future prospects. J. Am. Ceram. Soc..

[3] Eames C (2015). Ionic transport in hybrid lead iodide perovskite solar cells. Nat. Commun..

[4] Rohatgi, A. et al. An improved understanding of efficiency limiting defects in polycrystalline CdTe/CdS solar cells. In *Conf. Record of the 22nd IEEE Photovoltaic Specialists Conference*, 962–966 (IEEE, New York, 1991).

[5] Waser R, Aono M (2007). Nanoionics-based resistive switching memories. Nat. Mater..

[6] Liu H (2012). Copper ion liquid-like thermoelectrics. Nat. Mater..

[7] Xiao C (2012). Superionic phase transition in silver chalcogenide nanocrystals realizing optimized thermoelectric performance. J. Am. Chem. Soc..

[8] Ishiwata S (2013). Extremely high electron mobility in a phonon-glass semimetal. Nat. Mater..

[9] Qiu PF (2014). Sulfide bornite thermoelectric material: a natural mineral with ultralow thermal conductivity. Energy Environ. Sci..

[10] Weldert KS (2014). Thermoelectric transport in Cu_7_PSe_6_ with high copper ionic mobility. J. Am. Chem. Soc..

[11] Snyder GJ (2004). Disordered zinc in Zn_4_Sb_3_ with phonon-glass and electron-crystal thermoelectric properties. Nat. Mater..

[12] Lu X (2013). High performance thermoelectricity in earth-abundant compounds based on natural mineral tetrahedrites. Adv. Energy Mater..

[13] Riess I (2003). Mixed ionic–electronic conductors-material properties and applications. Solid State Ion..

[14] Shi X, Chen L, Uher C (2016). Recent advances in high-performance bulk thermoelectric materials. Int. Mater. Rev..

[15] Chen N (2017). Biomimetic ant-nest ionogel electrolyte boosts the performance of dendrite-free lithium batteries. Energy & Environ. Sci..

[16] Brown DR, Day T, Caillat T, Snyder GJ (2013). Chemical stability of (Ag, Cu)_2_Se: a historical overview. J. Electron. Mater..

[17] Dennler G (2014). Are binary copper sulfides/selenides really new and promising thermoelectric materials?. Adv. Energy Mater..

[18] Hinderman, J. D. *Thermoelectric Materials Evaluation Program Annual Technical Report for Fiscal Years 1980/1981. *Report No. MMM-2331-0691 (U.S. Department of Energy, United States, 1981).

[19] Stapfer, G. & Garvey, L. *Progress Report No. 29 for a Program of Thermoelectric Generator Testing and RTG Degradation Mechanisms Evaluation. *Report No. DOE/ET/33003--T2 (U.S. Department of Energy, United States, 1979).

[20] Balapanov MKh (2004). Ionic conductivity and chemical diffusion in superionic Li_*x*_Cu_2–__*x*_S (0≤*x≤*0.25). Phys. Status Solidi b.

[21] Yakshibaev RA (1984). Ionic conductivity and diffusion in superionic conductor α-Cu_2−δ_Se. Fiz. Tverd. Tela.

[22] Allnatt AR, Lidiard AB (1993). Atomic Transport in Solids.

[23] Kim SI (2015). Dense dislocation arrays embedded in grain boundaries for high-performance bulk thermoelectrics. Science.

[24] Biswas K (2012). High-performance bulk thermoelectrics with all-scale hierarchical architectures. Nature.

[25] Joshi G (2008). Enhanced thermoelectric figure-of-merit in nanostructured p-type silicon germanium bulk alloys. Nano. Lett..

[26] Wagner C (1972). The thermoelectric power of cells with ionic compounds involving ionic and electronic conduction. Prog. Solid State Chem..

[27] Werner K, Wiegand S (2002). Thermal Nonequilibrium Phenomena in Fluid Mixtures.

[28] Yokota I (1953). On the electrical conductivity of cuprous sulfide: a diffusion theory. J. Phys. Soc. Jpn..

[29] Korte C, Janek J (1997). Nonosothermal transport properties of *α*-Ag_2+δ_S: partial thermopowers of electrons and ions, the soret effect and heats of transport. J. Phys. Chem. Solids.

[30] Chakrabarti DJ, Laughlin DE (1983). The Cu–S (copper–sulfur) system. J. Phase Equilibria.

[31] Lukashev P, Lambrecht WRL (2007). Electronic and crystal structure of Cu_2*−x*_S: full-potential electronic structure calculations. Phys. Rev. B.

[32] Massalski, T. B., Okamoto, H., Subramanian, P. R., & Kacprzak, L. *Binary Alloy Phase Diagrams* 2nd edn (ASM International, Materials Park, OH,1990).

