# Supplementary material for: Suppression of atom motion and metal deposition in mixed ionic electronic conductors
Source: Nat Commun. 2018 Jul 25;9:2910. doi: 10.1038/s41467-018-05248-8 (PMC6060128; doi:10.1038/s41467-018-05248-8)
Supplement: Supplementary file 1 — Supplementary Information [file 41467_2018_5248_MOESM1_ESM.pdf]

Supplementary Information

Qiu, et al. Suppression of atom motion and metal deposition in mixed ionic/electronic conductors. Nat. Comms. (2018).

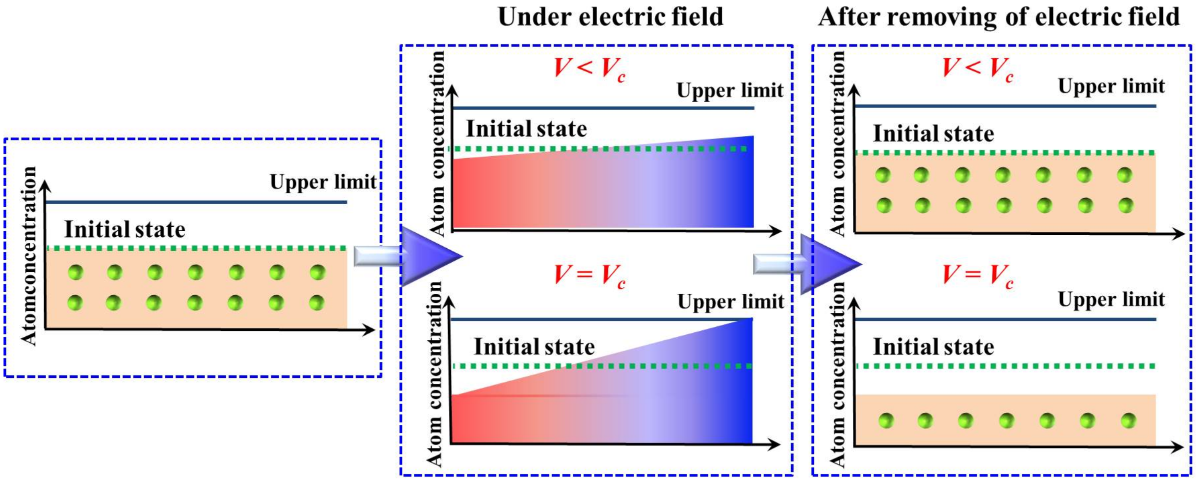

Supplementary Figure 1 Schematic material behavior under experimental conditions leading to the determination of the critical voltage, V_c.

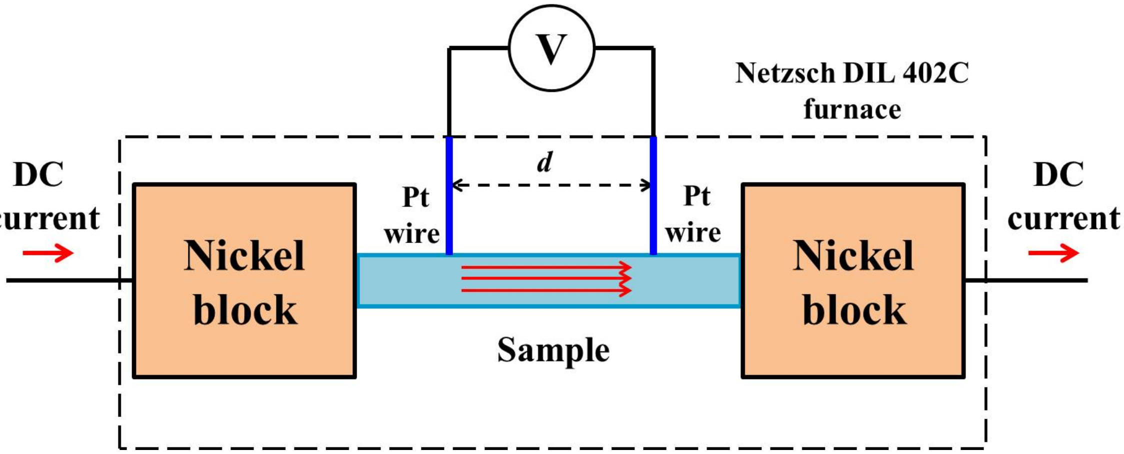

Supplementary Figure 2 Sketch map of the critical electric potential difference measurement apparatus in isothermal case.

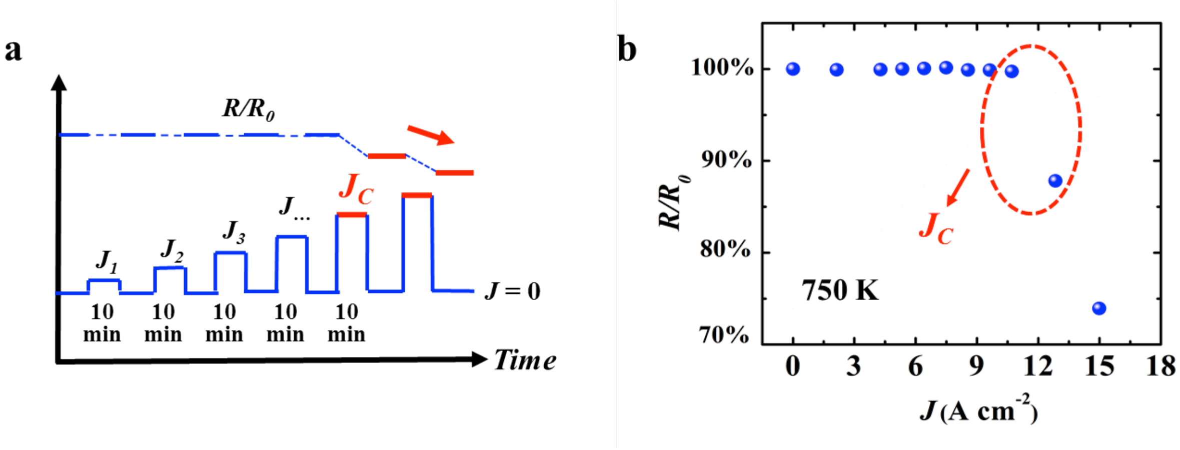

Supplementary Figure 3| Finding critical current density from resistivity measurements. (**a**) Sketch map of the measurement procedure for the critical electric potential difference in the isothermal case. The red lines depict the case when Cu deposits. (**b**) Current density dependence of the variation of relative electrical resistance for the $Cu_{1.97}S$ sample at 750 K. The points in the red circle depict the case when Cu deposits.

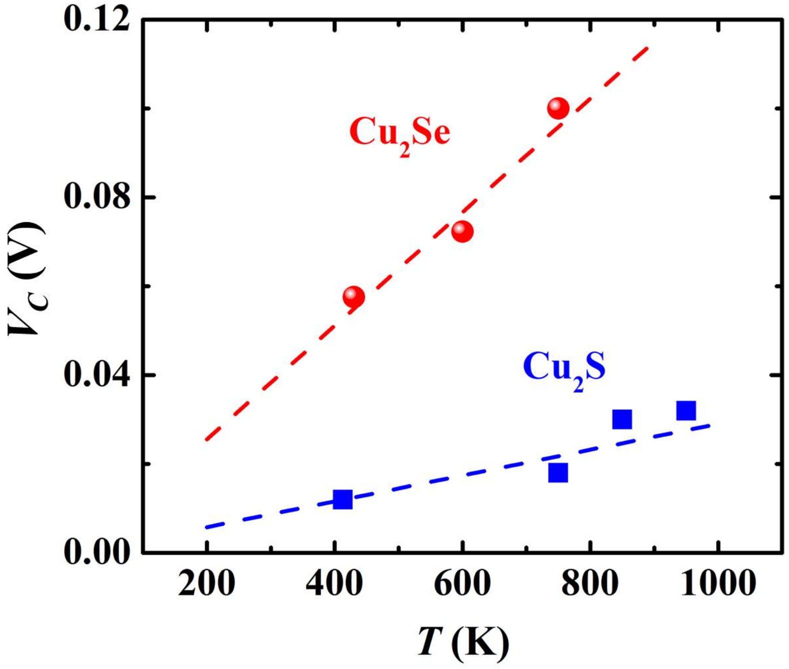

Supplementary Figure 4. Experimental critical electric potential difference for Cu_2Se and Cu_2S superionic phases at different temperatures. The dashed lines are guides to the eyes.

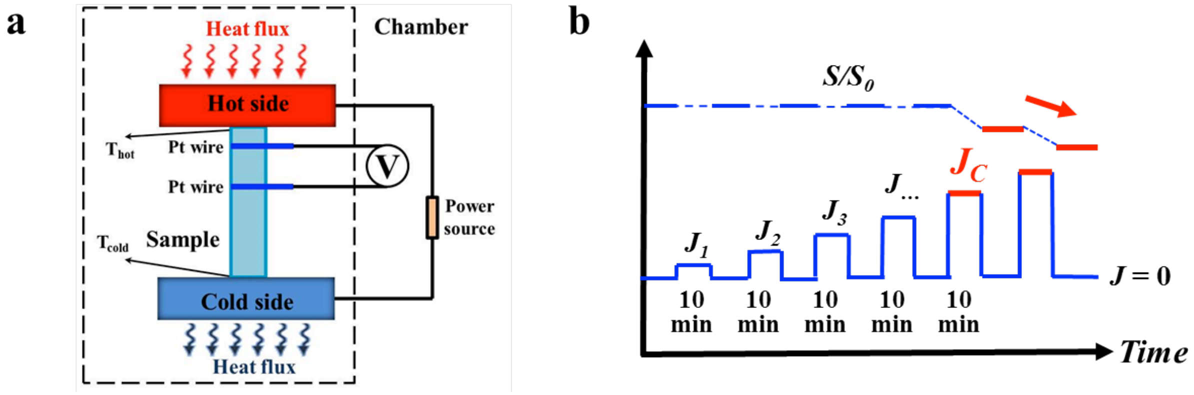

Supplementary Figure 5| Non-isothermal characterization. (**a**) Sketch map of the critical electric potential difference measurement apparatus in temperature gradient. (**b**) Sketch map of the measurement process for the critical electric potential difference in temperature gradient. The red lines depict the case when Cu deposits.

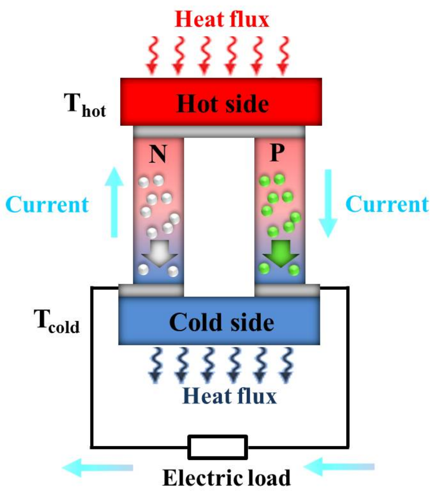

Supplementary Figure 6 Sketch map of the operating condition of a TE generator device.

Supplementary Table 1 Experimental critical current density J_c, critical electronic potential difference V_c, and electrical conductivity σ for several typical Cu-based TE MIECs at 750 K. The compositions determined by electron probe microanalysis (EPMA) are also included.

Composition	EPMA composition	Sample length	J_c (A cm^{-2})	V_c (V)	σ (10^4 S m^{-1})
Cu_2Se	$Cu_{2.004}Se$	10 mm	18-21	0.103(±0.008)	1.74
$Cu_2S_{0.5}Se_{0.5}$	$Cu_{1.990}S_{0.5}Se_{0.501}$		7-8	0.096 (±0.006)	0.52
Cu_2S	$Cu_{2.009}S$		0.14-0.21	0.024(±0.003)	0.02
$Cu_{1.99}S$	$Cu_{1.992}S$		0.47-0.57	0.053(±0.005)	0.14
$Cu_{1.97}S$	$Cu_{1.975}S$		11-13	0.087(±0.004)	1.14
$Cu_{1.96}S$	$Cu_{1.961}S$		18-20	0.090(±0.005)	2.01
$Cu_{1.94}S$	$Cu_{1.939}S$		27-29	0.093(±0.003)	2.86
$Cu_{1.90}S$	$Cu_{1.904}S$		51-54	0.107(±0.003)	4.67
$Cu_{1.97}S$	$Cu_{1.975}S$	6 mm	16-18	0.090(±0.005)	1.74
$Cu_{1.97}S$	$Cu_{1.975}S$	3 mm	28-32	0.093(±0.007)	0.52

Supplementary Table 2 The average electrical conductivity of $Cu_{1.97}S$ at different temperature ranges.

	350–473 K	350–523 K	350–573 K	350–623 K	350–673 K
σ_{avg} (10^4 S m^{-1})	1.01	0.96	0.94	0.98	1.03

Supplementary Note 1

Detailed electric potential difference equation derivation

The thermodynamic theory developed herein has some limitations. Firstly, it requires the stoichiometric mixed-ionic/electronic-conductor to be an intrinsic semiconductor with the molar fraction of holes (x_p) equaling to that of electrons (x_n). In this case, the off-stoichiometry δ can be associated with the holes/electrons by the relation δ = (x_p - x_n)/z, where z is the charge number of the cations. Secondly, samples with different off-stoichiometry should have the same structure as we use an ideal solution model for defects (vacancies and interstitial ions). Deviations from these requirements will lead to different mathematical forms for the microscopic theory developed herein.

The local equilibrium approximation of linear non-equilibrium thermodynamics states that, at steady state, the change in chemical potential of atom a is defined by the sum of changes in the electrochemical potentials of the electronic and ionic constituents,

$$\nabla\mu_a = \nabla\tilde{\mu}_e + \nabla\tilde{\mu}_i\,. \qquad (1)$$

The flux equation from linear non-equilibrium thermodynamics, for any charged species of particles (mol m^{-2}s^{-1}), is given by:

$$j = -\mathcal{L}\left[\nabla\tilde{\mu} + \left(\mathscr{s} + \frac{Q}{T}\right)\nabla T\right], \qquad (2)$$

where the coefficient, $\mathcal{L}$, obeys the Onsager reciprocity relations, and is related to the conductivity, σ, of the species by

$$\mathcal{L} = \frac{\sigma}{(zF)^2}\,, \qquad (3)$$

and z specifies the sign/magnitude of the charge. Then the current flux density (C m^{-2}s^{-1}) contributed by that species is

$$J = zFj = -\frac{\sigma}{zF}\left[\nabla\tilde{\mu} + s\,\nabla T\right], \qquad (4)$$

and we have used the convenient shorthand $s = \left(\mathscr{s} + \frac{Q}{T}\right)$, where $\mathscr{s}$ and Q/T have units of entropy, $\mathscr{s}$ is the specific entropy, and Q is the so-called heat of transport.[26,27] This equation can then be applied to any charged species, and the goal

is to define $\nabla\tilde{\mu}$ for both the ionic and electronic constituents in Eq. 1. Considering the case for the electronic carrier,

$$J_e = -\frac{\sigma_e}{z_e F}\left[\nabla\tilde{\mu}_e + s_e\,\nabla T\right], \qquad (5)$$

we can rearrange to find

$$\nabla\tilde{\mu}_e = -s_e\,\nabla T - z_e F\,\frac{J_e}{\sigma_e}. \qquad (6)$$

Considering the case for the ionic carrier, from Eq. 4, explicitly,

$$J_i = -\frac{\sigma_i}{z_i F}\left[\nabla\tilde{\mu}_i + s_i\,\nabla T\right] = 0 \qquad (7)$$

when ion-blocking electrodes are used (i.e., there can be no ion flux). Therefore the total electrochemical potential is the open circuit electrochemical potential,

$$\nabla\tilde{\mu}_i = (\nabla\tilde{\mu}_i)_{J_e=0} = -s_i\,\nabla T. \qquad (8)$$

Now it is possible to construct the steady-state chemical potential gradient of the neutral atom as

$$\nabla\mu_a = \nabla\tilde{\mu}_e + \nabla\tilde{\mu}_i = -z_e F\,\frac{J_e}{\sigma_e} - s^*\,\nabla T\,, \qquad (9)$$

where $s^* = s_e + s_i$. Considering transport in only the *x*-direction, $\nabla = \mathrm{d}/\mathrm{d}x$ and Eq. 9 can be rearranged and integrated by $\mathrm{d}x$, so that

$$\frac{J_e\,L}{\sigma_e} = -\frac{1}{z_e F}\Delta\mu_a - S^*\Delta T. \qquad (S10)$$

Here we use J_e for electronic current density (J in the main text), $\mathrm{d}x = L$, where L is the linear distance between the electrodes (nominally the sample length), and use an average $S^* = s^*/z_e F$.

Supplementary Methods

Critical electric potential difference measurements in isothermal case

The sketch map of the critical electric potential difference measurement apparatus in isothermal case is shown in Supplementary Figure 1. Two nickel blocks ($\Phi 10\times5$ mm^3) are used as the electrodes to conduct the electric current flowing through the measured samples. These high thermal-conductive nickel blocks can also weaken the temperature variation on the sample caused by the Peltier effect when stressing current on the sample. Two Pt wires are pasted on the sample to record the potential variation induced by the DC current. The electrodes, sample, and Pt wires are packaged into the furnace chamber of the Netzsch DIL 402C equipment. All measurements are carried out in static Ar atmosphere.

In order to simplify the experiments, we use nickel blocks as Cu ion blocking electrodes. The external electric field is generated by drawing a constant current across the sample. The magnitude of the build-up electric potential difference along the sample can be adjusted via tuning the value of current density. In order to sufficiently polarize the sample by diffusion of Cu atoms, the duration for each current pulse is 10 minutes. After switching off the current, we wait for 10 minutes to let the Cu atoms sufficiently diffuse back and reach again equilibrium. Then, the electrical resistance R is measured by using a 4-point method and a small DC current. The measured resistance R is then compared with the initial value R_0 before the next current pulse. If R is unchanged, the current density J is enhanced to further raise the electric potential difference across the sample, followed by repeating the above measurement processes. In this way, the critical current density and critical electric potential difference corresponding to the onset of Cu-metal deposition can be identified as the case when R shows an abrupt decrease (see Supplementary Figure 3a). Because we use a 4-point resistance measurement, the electric potential difference reported here does not include any potential drop across the electrodes where the Cu-metal deposition may or may not occur. Thus even if there is an over-potential drop across the electrodes to deposit Cu this potential drop is not included

in the electric potential difference measured here. Take $Cu_{1.97}S$ as an example, Supplementary Figure 3b shows the measurement results at 750 K.

Critical electric potential difference measurements in a temperature gradient

The sketch map of the critical electric potential difference measurements in temperature gradient is shown in Supplementary Figure 5a. The measured sample is located between a Cu block and a heater inside a nickel block. Good thermal contact is maintained at the interfaces using one compression spring. The Cu block is used as the cold side and its temperature is controlled by the circulating water. A program based on LabView software is used to control the input power to the heater. Two Pt wires are pasted on the sample near the hot end to record the Seebeck coefficient variation (S/S_0) induced by the temperature gradient and the DC current. The measurement is conducted inside a chamber that is filled with argon. This apparatus allows for simultaneous measurement of electric potential and temperature and application of current of up to 2 A. The length of the measured sample is 6 mm.

When the temperature at the hot side is raised to the specified value, we wait for two hours to let the sample reach the stationary state. After the initial Seebeck coefficient S_0 (= $V_0/\Delta T$, where V_0 and ΔT are the electropotental and the temperature difference between the two Pt wires, respectively) is recorded, a constant current is stressed on the sample. In order to sufficiently polarize the sample by diffusion of Cu atoms, the duration for each time current stress is 10 minutes. After switching off the current, we wait for 10 minutes to let the Cu atoms sufficiently diffuse back and reach again equilibrium. Then, the Seebeck coefficient S is recorded again and compared with the initial value S_0 before the next time current test. If S is unchanged, the current density J is increased to further raise the electric potential difference across the sample, followed by repeating the above measurement processes. In this way, the critical current density corresponding to the onset of Cu-metal deposition can be identified as the case when S shows an

abrupt decrease (see Supplementary Figure 5b). The critical electric potential difference can then be calculated. Similar with the above measurements in isothermal case, the over-potential drop across the electrodes is also not included here.
